# Electrochemical Sensor for Levosulpiride Detection and Its Adsorptive Removal from Wastewater

**DOI:** 10.1021/acsomega.4c11218

**Published:** 2025-04-23

**Authors:** Mohsin Javed, Afzal Shah, Iltaf Shah

**Affiliations:** † Department of Chemistry, Quaid-i-Azam University, Islamabad 45320, Pakistan; ‡ Department of Chemistry, College of Science, United Arab Emirates University, P.O. Box 15551, Al Ain, United Arab Emirates

## Abstract

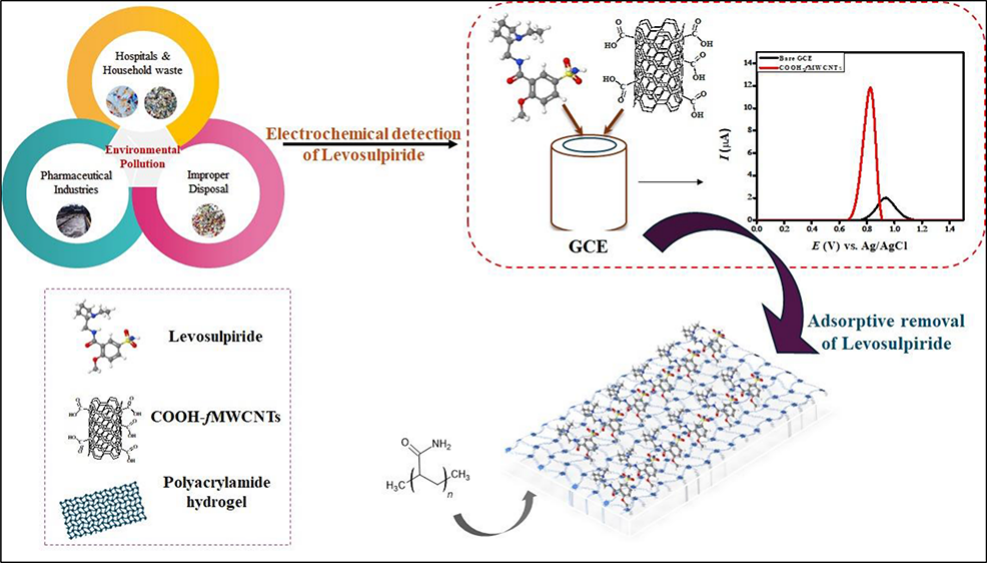

There is increasing apprehension regarding the harmful impacts of synthetic drugs, particularly the antipsychotic levosulpiride. Consequently, it is both an environmental and a social responsibility to create effective sensors and materials for the early detection and removal of this drug before consumption of contaminated water. The detection goal is achieved by the development of a highly sensitive and selective sensing platform, while the removal objective is achieved by the polyacrylamide hydrogel. The present study introduces a novel combination of advanced materials and green chemistry concepts for the development of an electrode modifier (functionalized MWCNTs) and an adsorbent (polyacrylamide hydrogel). Unlike conventional methods, where drug molecules slowly diffuse to the electrode surface, our technique for sensor preparation directly immobilizes these molecules on the electrode, leading to robust electrochemical signals and establishing a highly sensitive detection platform with a significantly reduced limit of detection (0.7 nM). Furthermore, while traditional adsorption processes may take hours or even days, our unique adsorbent for levosulpiride demonstrates effective removal in just 45 min, as confirmed by our experimental findings. Results revealed that adsorption occurred in accordance with the Langmuir model, while the kinetics of adsorption adhered to pseudo-second order kinetics. Thermodynamic parameters such as negative Δ*G* and positive Δ*S* revealed the spontaneous nature of the entropy-driven process of adsorption.

## 1 Introduction

The sixth objective of the United Nations’ sustainability agenda emphasizes the importance of ensuring clean and accessible water for everyone.^1^ The enormous rise in population has led to an increase in the discharge of effluents into the water reservoirs. Water pollution is caused by various industries (pharmaceutical, metal plating, leather, agricultural, iron and steel, and cosmetic) and household wastes. However, the pharmaceutical effluents are the main cause of concern^2,3^ because traditional methods of water purification cannot effectively remove these pollutants from wastewater. Pharmaceuticals are important to maintain human health; however, they are not free from adverse effects.^4^ These toxic effluents originate from pharmaceutical industries, hospitals, human excretion, and domestic sewage. Although these effluents are in concentrations of ng/L to mg/L, they have enormous adverse effects even at that concentration.^5^ Due to the current pandemic and rising population, the release of pharmaceutical effluents has increased substantially, which has led to a drastic rise in water pollution.^6^ Different pharmaceuticals show different solubilities and stabilities in water. Some of the drugs are highly toxic, such as antipsychotics, and they remain in aquatic environments for long periods.^7^ Because of their stability, the detection and treatment of these compounds in conventional water treatment plants is impossible. Additionally, antipsychotic drugs can interact with soil and water surfaces, making it essential to develop methods for the trace-level detection of pharmaceutical residues.^8^

Levosulpiride is an antipsychotic drug that is also used as a prokinetic agent. It is produced from sulpiride, which is a modified benzamide compound. Sulpiride’s levo-enantiomer was created for its antipsychotic effects, which are attributed to its ability to block dopamine D2 receptors.^9^ Levosulpiride has been found to have distinct pharmacological characteristics that make it particularly efficient in treating gastrointestinal motility problems and some psychiatric illnesses, setting it apart from its racemic counterparts.^10^ Levosulpiride is an antipsychotic medication associated with significant health risks. Elevated prolactin levels can cause gynecomastia and menstrual irregularities alongside a heightened risk of neuroleptic malignant syndrome and severe extrapyramidal symptoms. Additionally, it may lead to considerable weight changes, metabolic issues, and heart function alterations, which can result in QT prolongation, hepatotoxicity, and fluid balance disturbances. Furthermore, some adverse effects may be more severe in individuals taking this medication compared to those on other antipsychotic treatments.^11^

Enhanced sensitivity and lower detection limits are achieved by using MWCNTs as electrode modifiers in the case of electrochemical sensing. This is because of their large surface area and exceptional electrical conductivity. Because of the high surface energy and van der Waals forces, MWCNTs tend to agglomerate.^12^ Functionalization of MWCNTs with different groups (carboxyl, amino, or hydroxyl) can enhance their solubility and selectivity toward various compounds. Carboxyl groups added to the surface of MWCNTs greatly improve their ability to disperse, promote interactions with drug molecules, and offer numerous active sites for binding.^13^ COOH-*f*MWCNTs, when incorporated into electrochemical sensor platforms, can identify minute quantities of medicinal substances in various environments. Therapeutic drug monitoring, drug abuse detection, and environmental pharmaceutical residue surveillance rely on this skill. In medical and ecological settings, COOH-*f*MWCNTs-based sensors are a priceless asset for drug safety and efficacy monitoring due to their low detection limits, fast response, and excellent stability.^14^ Polyacrylamide hydrogel effectively adsorbs levosulpiride due to its significant porosity and capacity to swell in aqueous environments, which provides an extensive surface area for optimal drug retention.^15^ The hydrogel’s structural characteristics facilitate the efficient binding of levosulpiride molecules, rendering it suitable for the removal of this pharmaceutical compound from water-based environments.

In this regard, the current document presents a method for the detection and adsorptive removal of levosulpiride from wastewater. Much research has been conducted for the effective detection of levosulpiride in human serum using detection methods like spectrofluorimetric, hydrophilic interaction liquid chromatography-tandem mass spectrometry (HILIC-MS), spectrophotometric, high-performance liquid chromatography (HPLC), fluorescence, colorimetric, and electrochemical biosensors.^16−22^ The square wave voltammetry (SWV) was employed for the electrochemical investigation of levosulpiride. This technique has several advantages over other comparative electrochemical techniques, such as cyclic voltammetry (CV) and differential pulse voltammetry techniques (DPV). SWV is twice as sensitive as CV and much faster than DPV, making it ideal for efficiently obtaining intense signals. Moreover, it enhances Faradaic current by minimizing capacitive current, resulting in a significantly improved signal-to-noise ratio.

**Scheme 1:**
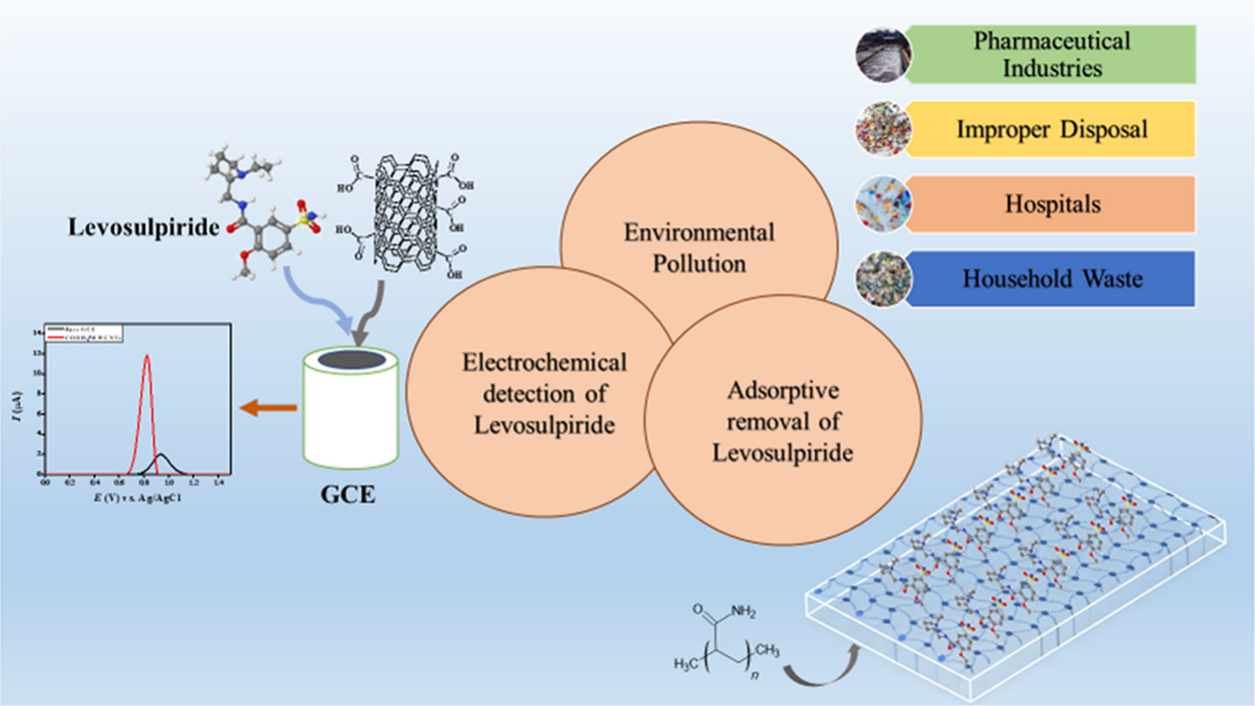
Schematic Illustration for the Electrochemical Detection and Adsorption of Levosulpiride

However, the literature review indicates that levosulpiride has not been electrochemically detected or eliminated from wastewater. Consequently, this research article aims to develop a sensitive sensing scaffold utilizing COOH-*f*MWCNTs for the electrochemical detection of levosulpiride, achieving a limit of detection of 0.7 nM, alongside effective adsorptive removal through the polyacrylamide hydrogel. This study presents the polyacrylamide hydrogel as an innovative approach for the adsorptive removal of levosulpiride. Schematics of the sources and techniques for achieving the objectives of levosulpiride detection and removal are presented in Scheme 1.

## 2 Experimental Section

### 2.1 Chemicals and Reagents

Sodium chloride (>99%), sodium dihydrogen phosphate monohydrate (>99%), sulfuric acid (98%), and disodium hydrogen phosphate dihydrate (>99%) were purchased from Merck. Ethanol (99.9%), potassium chloride (99%), potassium hydroxide (99%), and pristine MWCNTs (>95%) were purchased from Sigma-Aldrich. Sodium hydroxide (>97%) was purchased from Fluka, and hydrochloric acid (37%) and DMF (99%) were purchased from Riedel-deHaen. Levosulpiride (analytical grade) was purchased from StandPharm.

### 2.2 Synthesis of COOH-*f*MWCNTs

**Scheme 2:**
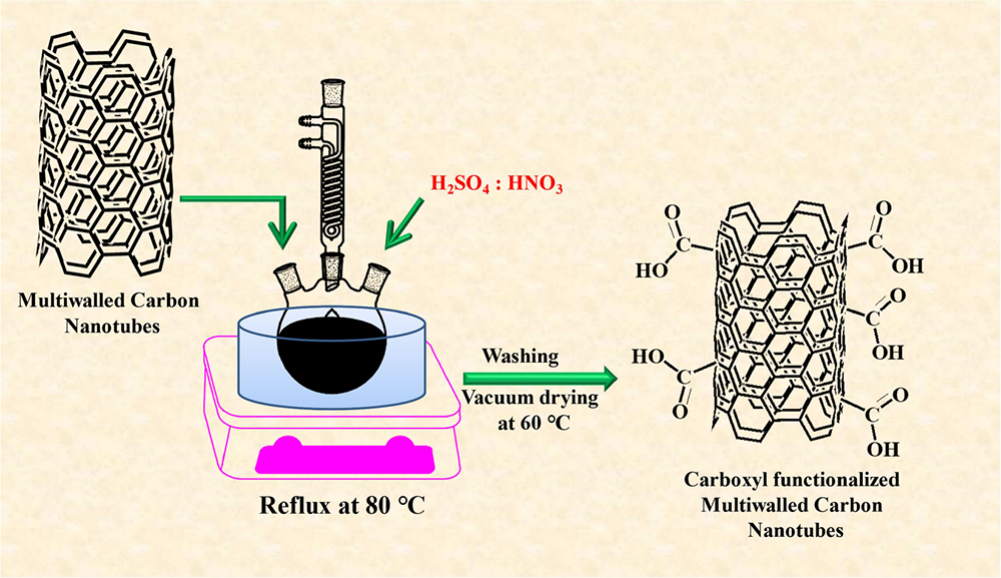
Synthesis Scheme for the Functionalization of MWCNTs

The synthesis of COOH-*f*MWCNTs from the pristine MWCNTs was carried out using the reported literature.^23^ The pristine MWCNTs were chemically functionalized by using strong acids. The acid solution containing H_2_SO_4_:HNO_3_ in a ratio of 3:1 was placed in a beaker. A weighed amount (1.5 g) of pristine MWCNTs was taken in a three-neck round-bottom flask, to which an acid solution was added, and the mixture was refluxed at 80 °C for 2 h. After adding some deionized water, the resulting mixture was kept for sonication for up to 2 h. Subsequently, the COOH-*f*MWCNTs were washed with double-distilled water until the pH became neutral, and the sample was filtered with membrane filter paper. After filtration, the COOH-*f*MWCNTs were kept in an oven at 60 °C for drying. The dried COOH-*f*MWCNTs were then collected and preserved in a sealed vial for future applications. Scheme 2 presents the synthesis scheme for preparing COOH-*f*MWCNTs using pristine MWCNTs.

### 2.3 Synthesis of the Polyacrylamide Hydrogel

**Scheme 3:**
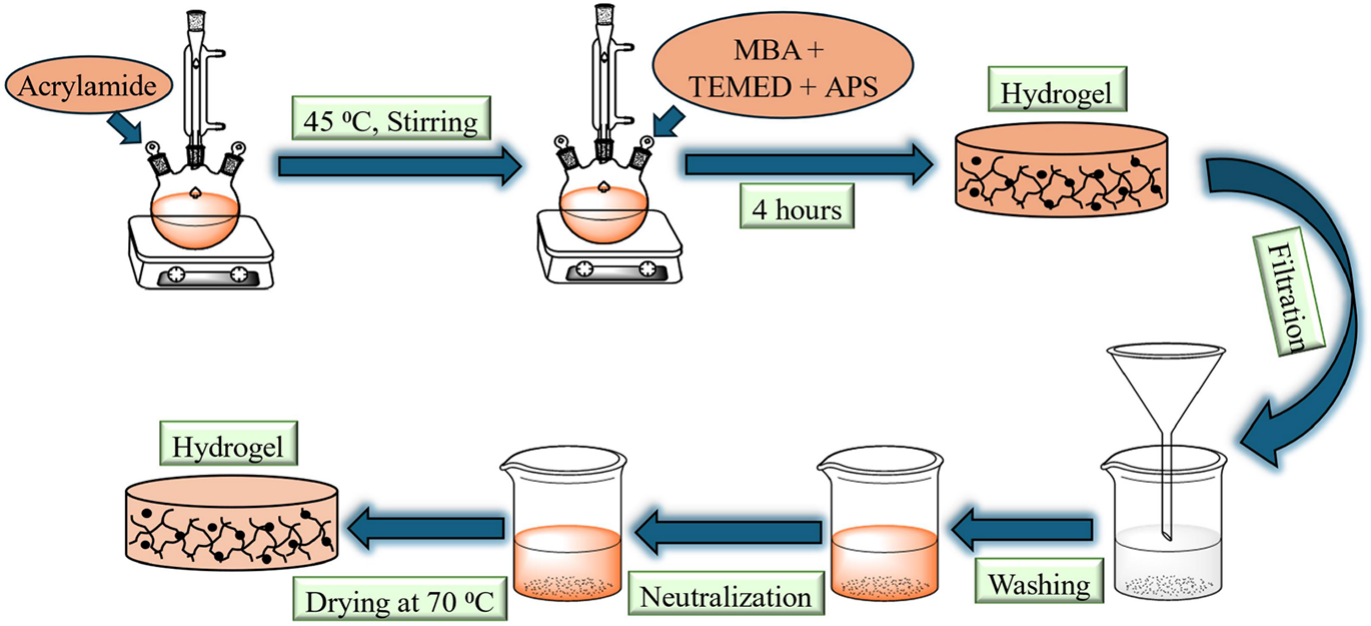
Synthesis Scheme for the Polyacrylamide Hydrogel

Free radical polymerization was employed to synthesize polyacrylamide hydrogel.^24^ In general, the experimental setup consisted of a flask fitted with a condenser and an argon inlet placed in an oil bath. First, in a 100 mL round-bottom flask, a (1 g/mL) acrylamide solution was heated at 45 °C and stirred. The mixture was purged for 15 min with argon to ensure an oxygen-free environment. After that, 1.25 mL of a solution (1%) of *N*,*N*′-methylene bis acrylamide was added to the mixture. To initiate the polymerization, 1 mL of ammonium persulfate (5 g/100 mL) as an initiator and 1.25 mL of tetramethyl ethylenediamine (1%) as an accelerator were added to the reaction mixture. The hydrogel was formed within 5 min, but the reaction continued for two more hours. The prepared hydrogel was dried in an oven at 50 °C for 24 h. The hydrogel particles were washed 5 times with acetone and DI water to remove impurities and unwanted species. After being washed, the microgel particles were neutralized with NaOH overnight. The particles were again washed with excessive acetone and deionized water. Ultimately, hydrogel particles were dried in a vacuum oven at 70 °C. The overall synthesis scheme can be seen in Scheme 3.

### 2.4 Electrode Modification

The glassy carbon electrode (GCE) surface with its sp^2^-hybridized carbon conjugated ring structure possesses a variety of functionalities, including aldehydes, carboxylic acids, alcohols, quinones, lactones, and ketones. The GCE was subjected to a cleaning procedure that incorporated both physical and chemical methods. It was scrubbed on a nylon mat soaked with a 1 μM aqueous alumina particle slurry. After this step, the transducer underwent sonication for 10 min in a mixture of ethanol, water, and acetone, followed by air drying. The electrochemical cleaning of the GCE involved conducting cyclic voltammograms within a potential range of −0.1 to 1.0 V until consistent and reproducible blank voltammograms were achieved.

Electrophoretic deposition for coating applications provides effective control; however, it is more time-consuming and suitable only for conductive substrates. This method produces uniform thin films but results in material waste. While sputtering is a slower and more costly technique, it ensures strong adhesion and uniform film thickness across surfaces. Chemical vapor deposition yields high-purity coatings but involves the use of hazardous substances. Lastly, electroplating is a cost-effective and rapid process, although it may introduce impurities and prolong the thickness buildup. The drop-cast method was employed to effectively deposit the modifier and analyte onto the surface of the GCE. This method is also helpful, as we can control the number and size of droplets, resulting in uniform deposition of the sensing materials. The method is cost-effective, as only a droplet sample of the analyte is sufficient to record its voltammetric signals. Additionally, it is a straightforward technique that does not require any complex procedure and allows for better control and generation of intense signals owing to the closer accessibility of the analyte to the transducer surface. The other techniques used for electrode deposition are also discussed in the revised manuscript to highlight the preference of our electrode modification method.

Voltammograms were recorded on Metrohm Autolab serial number MAC80146 (galvanostat/potentiostat) from Utrecht, The Netherlands. Electrochemical impedance spectroscopy (EIS) was conducted on a Gamry Interface 5000E Potentiostat (Louis Drive, Warminster, PA 18974, USA). The analysis utilized Gamry Framework version 7.9.0 along with Gamry Echem Analyst version 7.9.0 software.

To modify the GCE, a COOH-*f*MWCNTs dispersion was created in dimethylformamide through ultrasonication for 5 h at 1 mg per 1 mL. The GCE underwent a precleaning process before being modified with COOH-*f*MWCNTs using a layer-by-layer technique. Electrochemical experiments were conducted using a 0.1 mM solution of levosulpiride. The analyte solution was applied through drop casting and allowed to dry on the modified electrode’s surface. Following this, the electrode was immersed in the supporting electrolyte to perform voltammetric experiments.

## 3 Results and Discussion

### 3.1 Characterization of MWCNTs and COOH-*f*MWCNTs

**Figure 1:**
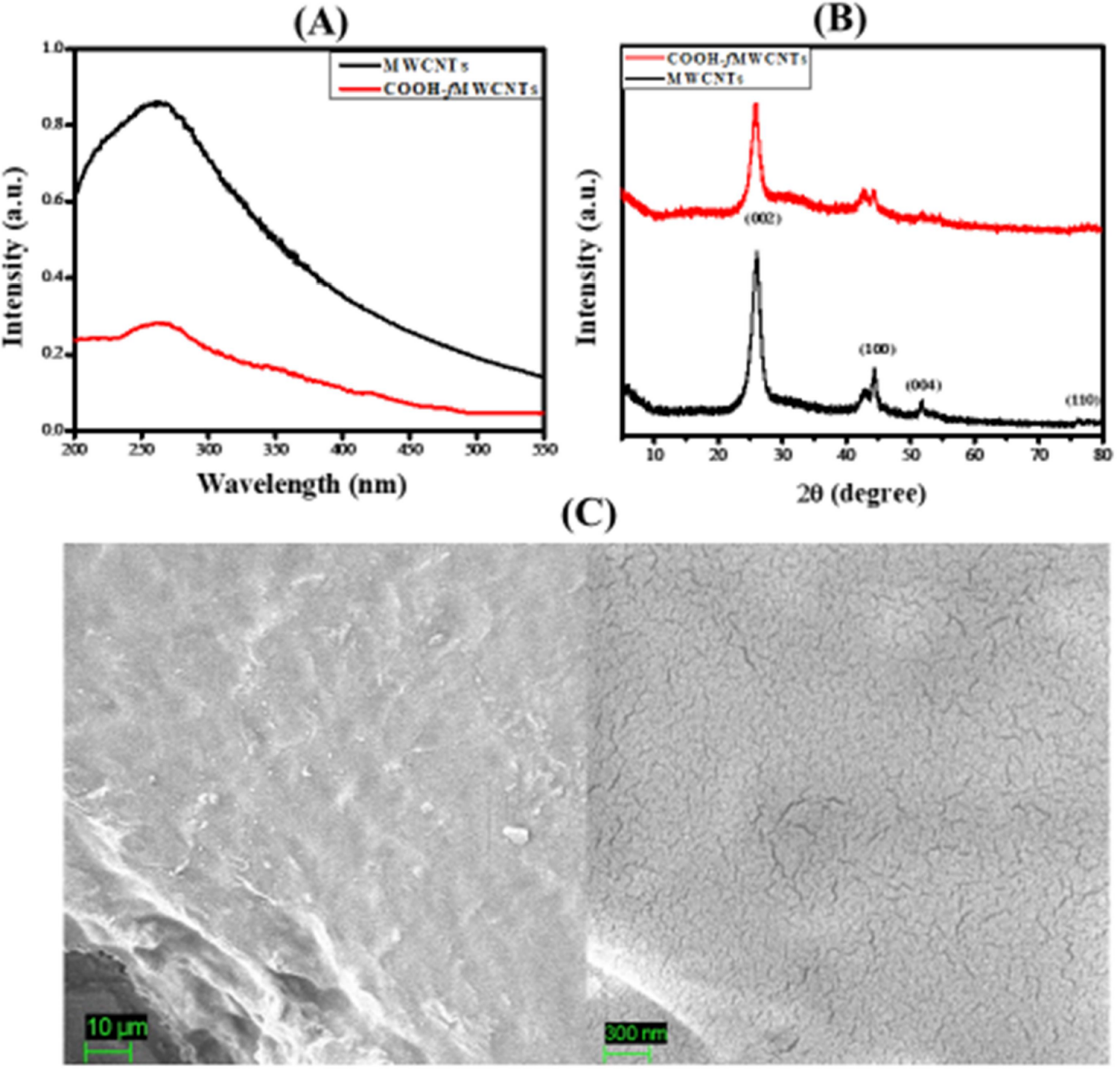
(A) UV–vis spectra of MWCNTs and COOH-*f*MWCNTs (B) XRD analysis of MWCNTs and COOH-*f*MWCNTs (C) SEM micrographs of the polyacrylamide hydrogel at different resolutions.

The optical properties of MWCNTs and COOH-*f*MWCNTs were examined by UV–vis spectroscopy. The MWCNTs and COOH-*f*MWCNTs were dispersed in distilled water and sonicated for 30 min before recording UV–vis spectra. The UV–visible absorption spectra of the functionalized MWCNTs are shown in Figure 1A. The observed signal in the range of 200–300 nm aligns with existing literature.^25^ The MWCNTs and COOH-fMWCNTs exhibited increased absorption peaks in the ultraviolet region. Concurrently, the absorbance progressively diminishes as one approaches the near-infrared region, a phenomenon likely due to scattering effects.

XRD analysis was conducted to investigate the structural characteristics of both pristine multiwalled carbon nanotubes (MWCNTs) and carboxylated functionalized MWCNTs (COOH-fMWCNTs). The detected signals at 2θ = 26.01° and 43.5°, corresponding to the (002) and (100) planes, respectively, align closely with JCPDS No. 41-1487, thereby confirming the presence of the hexagonal graphite structure in the MWCNTs. The diffraction peak at 2θ = 26.01° for pristine MWCNTs shifting toward 25.74° with a corresponding index plane of (002) indicates the graphite-like structure. The average crystallite size calculated according to the Debye–Scherrer formula^26^ for pristine MWCNTs and COOH-*f*MWCNTs is 4.22 and 4.29 nm, respectively. Functionalization of pristine MWCNTs with COOH groups did not affect their original tube-like morphology. The XRD diffractograms for COOH-*f*MWCNTs and MWCNTs are shown in Figure 1B. SEM was carried out to examine the surface morphology of the polyacrylamide hydrogel. SEM micrographs are presented in Figure 1C, supporting the successful synthesis of a polyacrylamide hydrogel.

**Figure 2:**
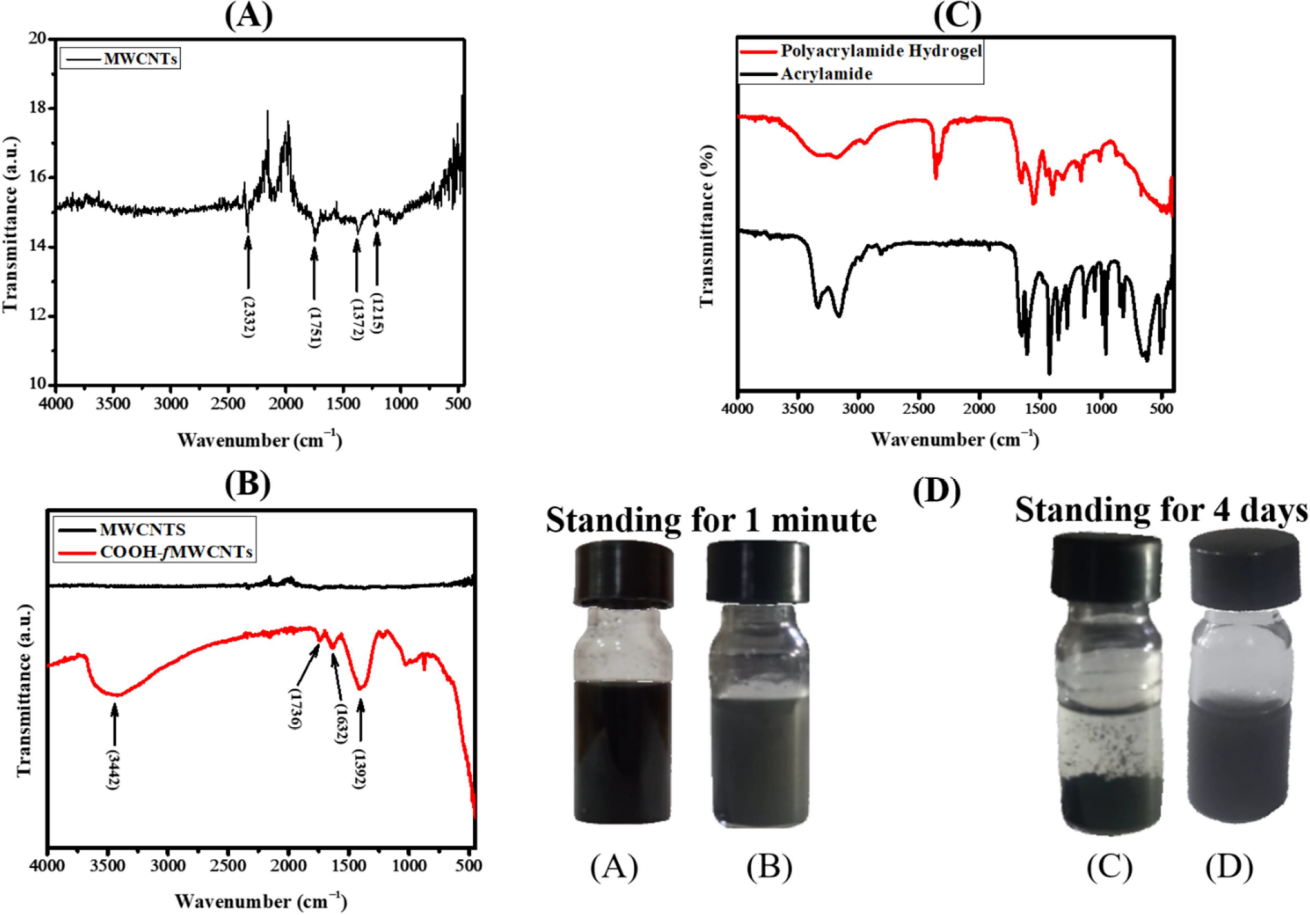
FTIR analysis of (A) pristine MWCNTs, (B) stack plot of MWCNTs and COOH-*f*MWCNTs, (C) polyacrylamide hydrogel and acrylamide, (D) dispersion test for the functionalization of MWCNTs [(A) and (C) MWCNTs, and (B) and (D) COOH-*f*MWCNTs].

The FTIR spectra of pristine MWCNTs are presented in Figure 2A. The signals at 2332, 1751, 1372, and 1215 cm^–1^ correspond to C–H_*x*_ and aromatic ethers. The peaks between 1300 to 1750 cm^–1^ indicate the apparent vibrational modes of MWCNTs. When the pristine MWCNTs were functionalized, the presence of functional groups was confirmed by comparing the FTIR spectra presented in Figure 2B. The broad peak at 3442 cm^–1^ ensures the presence of −OH stretching, while the signal at 1736 cm^–1^ can be attributed to the presence of carbonyl groups in the functionalized MWCNTs. The weak signal at 1632 cm^–1^ indicates the presence of traces of water.^27^

The FTIR spectra of the polyacrylamide hydrogel and acrylamide monomer are presented in Figure 2C, showing the characteristic peaks of all essential functional groups in the hydrogel. The double peak observed between 3100 and 3500 cm^–1^ in the spectrum of acrylamide is ascribed to the amide unit, which is absent in the spectrum of polyacrylamide hydrogel.^28^ The broad signal at 3188 cm^–1^ relates to the −NH stretching vibration from the amine groups (−CONH_2_). A shoulder peak at 2944 cm^–1^ originated from the symmetric and antisymmetric stretching vibrations of C–H bonds of the alkyl group. A peak at 2363 cm^–1^ could be attributed to atmospheric carbon dioxide, which interferes with the spectrum. It may correspond to some weak overtones or combination bands. The signal at 1659 cm^–1^ represents the stretching vibrations of the carbonyl group (C=O) in the amide functionality of polyacrylamide. The signal at 1545 cm^–1^ is attributed to the combination of C–H stretching and N–H bending vibrations. The peak at 1402 cm^–1^ may arise due to the coupling of C–N stretching vibrations with CH_2_ bending. The peak at 1163 cm^–1^ arises due to C–N stretching vibrations.

The functionalization of MWCNTs with different functional groups can be confirmed via various methods, and monitoring the dispersion of MWCNTs and functionalized MWCNTs is one of them.^23^ The dispersions of CNTs in DMF were made by taking 1 mg of CNTs in 1 mL of DMF in glass vials, followed by sonication for up to 30 min. After this, a suspension of CNTs was observed in glass vials. The pristine MWCNTs agglomerated because of their hydrophobic nature, high surface energy, and poor solubility can be observed in Figure 2D. On the other hand, the COOH-*f*MWCNTs, being electrostatically stable by the negative charges introduced by acid treatment, can form a stable suspension with improved dispersibility. Because of improved dispersion in the case of COOH-*f*MWCNTs, no appreciable change was observed, even after 4 days. This phenomenon can be attributed to the carboxylic functionalization of MWCNTs. Additionally, there is a slight color difference (black to lighter black) after the functionalization of carboxylic groups on pristine MWCNTs.

### 3.2 Electrochemical Characterization

**Figure 3:**
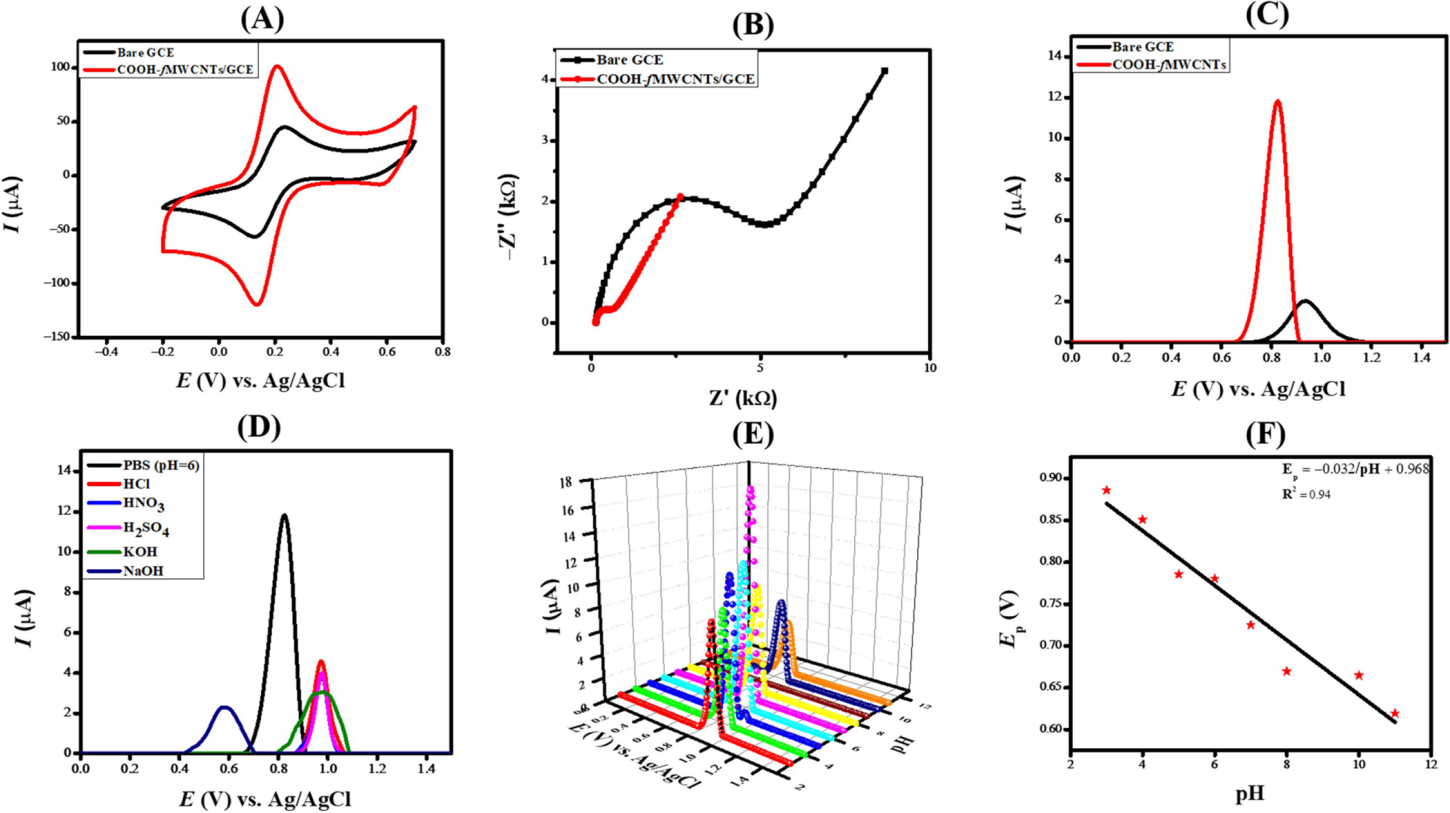
(A) CVs of the redox probe obtained at the bare and COOH-*f*MWCNTs/GCE. (B) Nyquist plots generated utilizing electrochemical impedance spectroscopy data collected at the bare and modified GCE in a solution of 5 mM K_3_[Fe(CN)_6_] and 0.1 M KCl, covering 1 Hz to 100 kHz frequency range. (C) SWVs of 100 μM levosulpiride at bare and COOH-*f*MWCNTs. (D) Influence of the supporting electrolyte on the square wave voltammetric response of levosulpiride. (E) Impact of the pH of the medium on the peak current intensity of levosulpiride (F) *E*_p_ vs pH plot.

CV was used to examine the electrocatalytic function of the electrode modifier. The CV experiments utilized 0.1 M KCl as an inert electrolyte and 5 mM K_3_[Fe(CN)_6_] as a redox probe. The results, illustrated in Figure 3A, indicate a notable enhancement in peak height at the COOH-*f*MWCNTs/GCE in comparison to the peak intensity at the bare GCE’s response to the redox probe. This increase in peak current, accompanied by a reduction in peak separation, highlights the electrocatalytic properties of the electrode modifier, facilitating efficient electron transfer and demonstrating the potential of the developed sensing platform for investigating redox events.

The surface area significantly impacts the sensing ability of the designed sensor. On the sensing scaffold’s higher surface area, more species exchange results in a higher current signal. Shifting the anodic peak to lower potentials pinpoints the electrocatalytic role of the electrode modifier. The greater surface area provides more active sites that lead to the exchange of more charge carriers and the consequent appearance of intense current signals. The Randles–Sevcik equation, which is employed to determine the electroactive surface area of an electrode, establishes a relationship between the peak current and variations in the scan rate. The scan rate is one of the factors that determine peak current for simple redox processes like ferrocene/ferrocenium pair, together with diffusional and concentration properties of the electroactive species.^29,30^
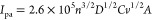
1

In the above, *I*_pa_ refers to the anodic peak current, while *n* indicates the number of electrons involved. The electroactive surface area of the electrode is denoted by *A* in cm^2^, *D* represents the diffusion coefficient in cm^2^ s^–1^, *v* signifies the scan rate in V s^–1^, and *C* denotes the concentration in mol cm^–3^. The *D* value for potassium ferricyanide is 7.6 × 10^–6^ cm^2^ s^–1^ and *n* = 1. Table S1 shows the electroactive surface area calculated through the Randles–Sevcik equation.

The COOH-*f*MWCNTs showed a maximum peak current of approximately 102 μA with a peak separation of 69 mV. The modified electrode exhibited a surface area that was approximately 2.5-fold increased compared to the bare electrode. The modified electrode exhibits functional groups and more sites for drug molecules to attach, resulting in faster electron transfer. The COOH-*f*MWCNTs/GCE exhibited maximum peak current with minimum peak separation, indicating its high conductivity compared to bare GCE.

Exchange current density (*J*_0_) serves as a vital metric in electrochemistry, representing the kinetics of electrochemical reactions under equilibrium conditions where there is no net current. A higher *J*_0_ indicates a more active electrode surface, facilitating faster redox processes. This characteristic is particularly important for sensing platforms as it directly affects their sensitivity and response time. An increased *J*_0_ indicates that the sensor is capable of detecting minute amounts of the analyte with greater effectiveness, thereby enhancing its sensitivity. The following equation is used for calculating the exchange current density
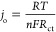
2

In the above equation, *R* symbolizes the general gas constant, *T* is temperature, and n is the number of electrons involved in a redox event, which is 1 for the K_3_[Fe(CN)_6_] redox probe, while *R*_ct_ is the charge transfer resistance. The greater surface area and faster electron transfer kinetics resulted in a higher *J*_0_ (55.84 μA/cm^2^) for COOH-*f*MWCNTs/GCE compared to bare GCE (5.51 μA/cm^2^).

EIS is a highly advanced analytical technique that allows the investigation of impedance in electrochemical systems over a wide range of frequencies. EIS offers comprehensive data regarding an electrochemical cell’s resistive and capacitive characteristics by measuring the current that results from introducing a pulse of alternating current voltage to the cell. This method provides a comprehensive understanding of the impedance, including both real and imaginary components. It allows for the detailed analysis of the interactions and electrochemical processes at the electrode–solution interface. EIS allows for a comprehensive analysis of sensor interfaces. The performance of the sensor is assessed from the double-layer capacitance, diffusional properties, and charge transfer resistance. Moreover, EIS is responsive to variations in impedance, resulting from alterations in the analyte concentration. The high level of sensitivity allows for the identification and measurement of the targeted analyte. EIS is also useful for assessing the stability and reliability of sensors over time, offering information about the durability of sensors.^31^

An EIS spectrum obtained in solution includes both semicircular and linear segments. The semicircular feature in the higher frequency domain represents the charge transfer resistance (*R*_ct_), whereas the linear portion resulting from diffusional transport is associated with the Warburg resistance (*Z*_w_). Both *Z*_w_ and *R*_ct_ are related to the mass transfer and electron transport phenomena taking place at the interface of the electrode and the electrolyte.^32^Figure 3B presents the Nyquist plots, where the larger semicircle indicates a higher resistance (*R*_ct_ value of 4653 Ω) to charge transport between the redox probe and the GCE. In contrast, the COOH-*f*MWCNTs/GCE demonstrate significantly lower resistance with an *R*_ct_ value of 459.7 Ω, highlighting its effectiveness as a highly conductive platform for efficient redox event sensing. Additionally, Figure S1 illustrates the application of Randle’s equivalent circuit to the impedance data, with the assessed EIS parameters detailed in Table S2.

### 3.3 Voltammetric Study of Levosulpiride

The sensitivity of the sensing platform for the electro-oxidation of levosulpiride was investigated by recording square wave voltammograms using PBS with a molarity of 0.1 M and pH 6, as depicted in Figure 3C. The SWVs were recorded at a deposition potential of 0.1 V and a deposition time of 5 s. Figure 3C illustrates the square wave voltammetric response of the bare and modified GCE toward the oxidation of 100 μM levosulpiride. It is clear from Figure 3C that COOH-*f*MWCNTs/GCE showed the maximum anodic peak current compared to bare GCE. It is evident from the CV and EIS data that COOH-*f*MWCNTs/GCE has a greater surface area and lower resistance to charge transport, making it an effective sensing platform for the electro-oxidation of levosulpiride.

COOH-*f*MWCNTs are promising for use as electrode modifiers in voltammetric sensing because of their high surface area and −COOH groups. Levosulpiride contains an amine group. COOH-*f*MWCNTs are suitable for the electrochemical sensing of drugs containing amine groups (−NH_2_). Amine-carboxylate bonding binds the drug molecule’s NH_2_ group to the carboxylic groups of the COOH-*f*MWCNTs’ carboxylic groups. This selective contact (electron donating and electron withdrawing) decreases sample interference. It concentrates the drug near the electrode, enhancing the electrochemical signal and possibly identifying low drug concentrations. So COOH-*f*MWCNTs/GCE was selected for the electrooxidation of levosulpiride owing to its high surface area and amine-carboxylate binding.

### 3.4 Optimization of Experimental Parameters

The composition of the electrolyte, pH level, deposition potential, and deposition duration can greatly influence the signal of the analyte. Consequently, various experimental parameters are fine-tuned to achieve a strong signal from the analyte at the sensing scaffolds. Optimization of the inert electrolyte is a crucial step in electrochemical sensing, as its quality can significantly impact the sensitivity of the sensing scaffold. Changing the supporting electrolyte can affect the conductivity, ionic strength, ion migration, and chemical equilibria. The primary objective of adding a supporting electrolyte is to enhance conductivity by minimizing the voltage drop (IR drop) within the system.^33^

The square wave voltammetric response for the electrochemical sensing of levosulpiride was investigated using various supporting electrolytes, including 0.1 M HNO_3_, PBS (pH 6), 0.1 M HCl, 0.1 M KOH, 0.1 M NaOH, and 0.1 M H_2_SO_4_. Figure 3D shows the influence of changing the supporting electrolyte on the oxidation signal of levosulpiride. The strongest signal for the analyte at the COOH-*f*MWCNTs/GCE was detected when PBS at pH 6 served as the inert electrolyte. Therefore, PBS was selected for further voltammetric analysis of levosulpiride, as evident from the bar graph in Figure S2A.

The detection of levosulpiride at COOH-*f*MWCNTs/GCE relies on the PBS electrolyte, underscoring the significance of pH optimization. Consequently, it is essential to conduct a carefully regulated pH experiment to reduce interference from the external environment. To assess the impact of pH, we explored the electrochemical sensing of levosulpiride (100 μM) in PBS across a pH range of 3–11. Figure 3E demonstrates how pH influences the oxidation of levosulpiride, whereas Figure S2B shows the variation in current relative to pH levels. An initial rise in peak current was noted from pH 3 to 7, after which a consistent decrease was recorded. The highest peak current was achieved at pH 7, which was identified as the optimal pH for subsequent electrochemical studies of levosulpiride.

**Figure 4:**
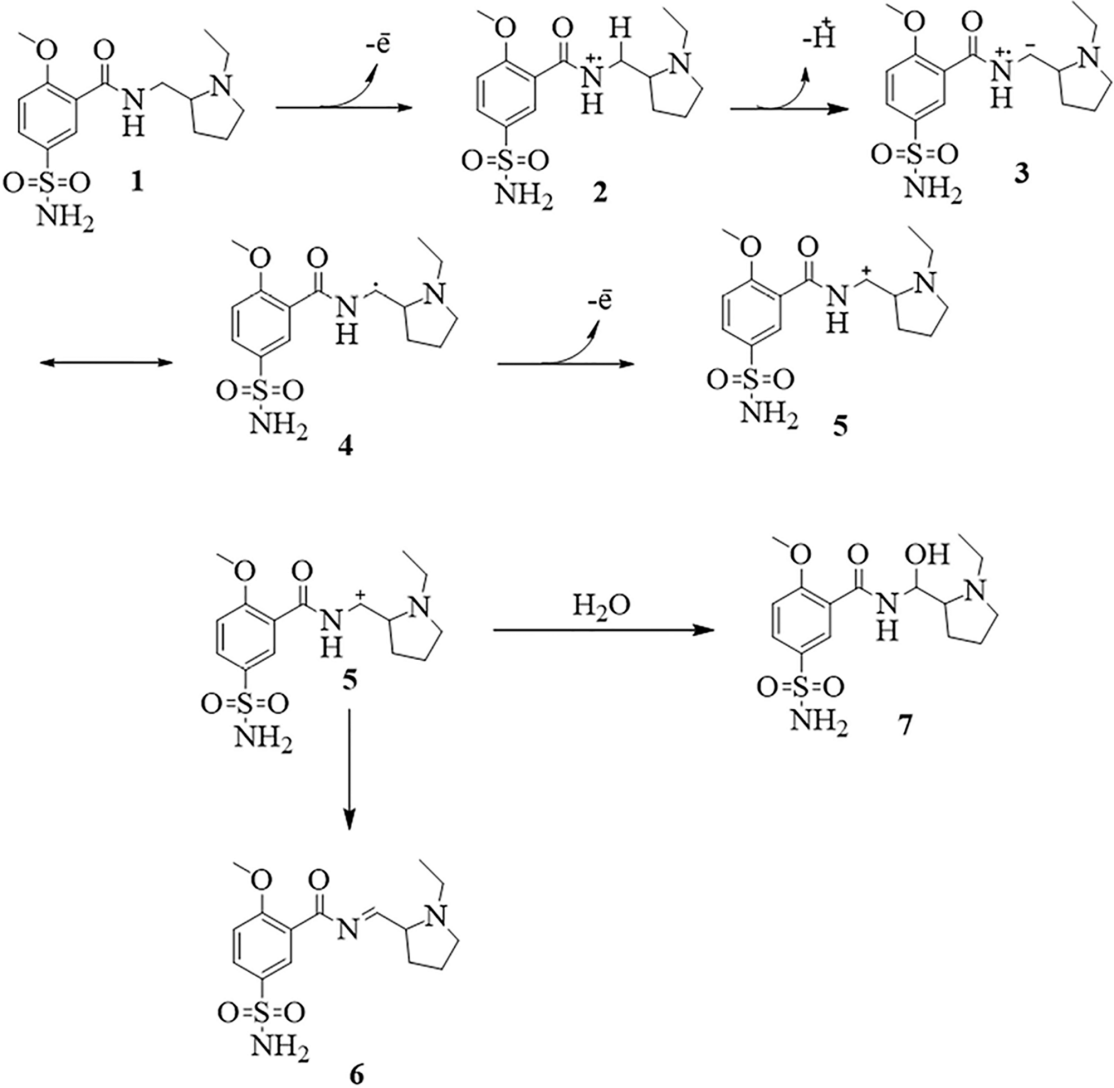
Proposed mechanism for the electrooxidation of levosulpiride.

It was observed that by changing the pH, the peak potential was also varied, confirming the participation of electrons and protons during the electrooxidation of levosulpiride. An *E*_p_ vs pH plot was created to assess the participation of electrons and protons. The observed shift in peak position with varying pH levels indicates the engagement of both electrons and protons in the electrooxidation reactions. Figure 3F illustrates the *E*_p_ vs pH plot. The number of electrons was quantified through the full width at half-maximum (fwhm), while the proton-to-electron ratio was derived from the Nernstian slope. The mechanism presented in Figure 4 was proposed by considering the experimentally obtained parameters and the established organic chemistry principles.

The deposition potential plays a crucial role in electrochemical sensing, impacting several key factors in the detection process. When a preconcentrated analyte is applied to the electrode surface, it increases sensitivity by enabling the sensing scaffold to identify lower concentrations of the drug. Furthermore, it enhances selectivity by preferentially depositing the analyte and minimizing interference from the environment, thereby ensuring the precision of the sensing scaffold.^33^ Moreover, an optimized accumulation potential helps stabilize the preconcentrated analyte by preventing side reactions or degradation. Bulky analyte groups can cause steric hindrance, making deposition on the electrode’s surface more difficult and impacting electrochemical behavior. Proper orientation of the analyte is necessary to ensure that electroactive groups are oriented toward the electrode surface during the redox process. Therefore, optimizing the deposition potential enhances the sensitivity, signal clarity, analyte deposition on the sensing scaffold, and overall efficiency.

The impact of deposition potential on the oxidation of levosulpiride was studied with the deposition potential varying from −0.2 to 0.2 V. There was a gradual increase in peak current as the deposition potential moved from −0.2 to 0 V, followed by a decrease in peak current beyond 0 V. Figure S2C illustrates the influence of the deposition potential on the electrooxidation of levosulpiride. The maximum anodic peak current was observed at a deposition potential of 0 V, where the maximum amount of the drug was accumulated on the electrode surface, leading to a strong interaction with the designed sensor. Therefore, 0 V deposition potential was selected for further analysis of levosulpiride. Figure S2D shows the variation in the anodic peak current as a function of deposition potential.

The duration of the deposition plays a critical role in the performance of the electrochemical sensing platform. Typically, an extended deposition time can improve the signal strength, as it allows for greater accumulation of the analyte on the electrode surface. Nonetheless, prolonged deposition may also result in diminished signal intensity due to potential saturation of the analyte and interference from the surrounding medium. Furthermore, inconsistent deposition times can compromise repeatability by leading to uneven distribution of the drug on the electrode. Thus, it is essential to optimize the deposition duration to ensure effective drug deposition, reduce nonspecific adsorption, and maintain reliable and precise outcomes. We investigated the effect of deposition time on the oxidation of levosulpiride, varying the duration from 5 to 20 s at a deposition potential of 0 V in PBS (pH 7) as the supporting electrolyte. An increase in peak current was observed as the deposition time increased from 5 to 10 s, followed by a decrease in peak current beyond 10 s. The maximum anodic peak current was observed at a deposition time of 10 s, as shown in Figure S2E. This indicates that, at this accumulation time, the drug is optimally oriented and accumulated on the electrode surface. The decrease in the peak current noted with longer deposition times can be attributed to the saturation of available sites on the modifier by analyte molecules. Figure S2F illustrates the variations in the peak current in relation to deposition time. The strongest response was recorded at a deposition time of 10 s and a deposition potential of 0 V, which prompted the selection of these parameters for further electrochemical investigations.

### 3.5 Analytical Application of the Designed Sensor

#### 3.5.1 Evaluation of the Limit of Detection and Quantification of Levosulpiride

**Figure 5:**
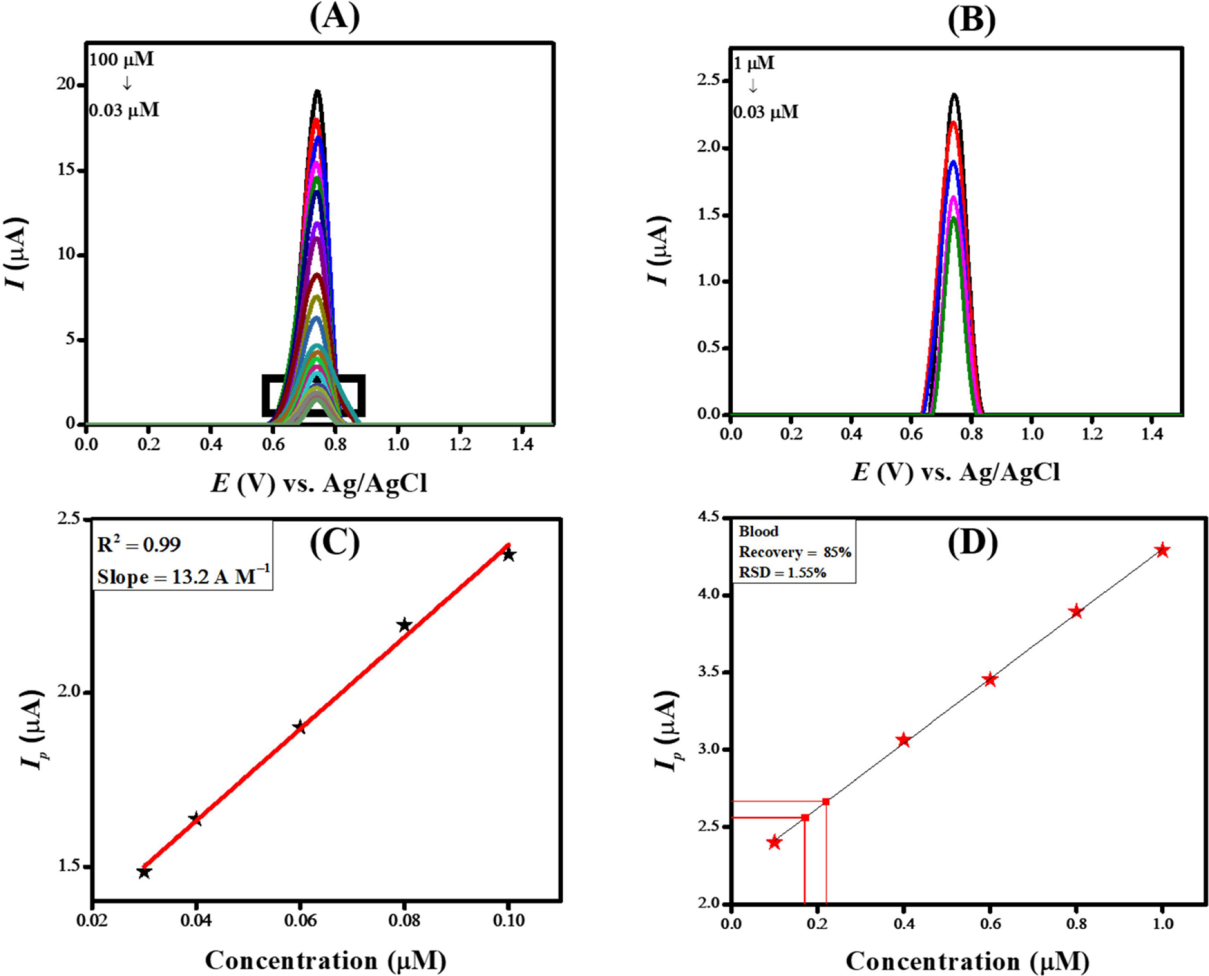
(A) Alteration of current in relation to concentration changes under preoptimized conditions for levosulpiride (0.03–100 μM), (B) voltammograms used for calibration plot (0.1–0.03 μM), (C) calibration plot at lower concentrations for levosulpiride, and (D) calibration plot showing recovery of levosulpiride in blood.

SWVs were recorded for different concentrations of levosulpiride under preoptimized conditions to evaluate the sensitivity of the developed sensor. The detection process involved varying the concentration of levosulpiride from 0.03 to 100 μM, as depicted in Figure 5A, with supplementary data for lower concentrations available in Figure 5B. The calibration curve is presented in Figure 5C. The limit of detection (LOD) and limit of quantification (LOQ) were determined using designated equations.^34^
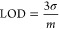
3
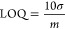
4Here, “*m*” represents the slope value derived from the calibration plot, while “σ” denotes the standard deviation obtained from the peak current value of the blank solution across 12 runs. The estimated LOD and LOQ for levosulpiride are 0.7 and 2.6 nM, respectively. Table 1 compares the analytical performance of various sensors used for levosulpiride detection.

**Table 1:** Comparison of the Analytical Performance of Reported Sensors with Our Designed Sensor for the Detection of Levosulpiride

method	analyte	linear range	LOQ	LOD	recovery	refs
HPLC-UV	levosulpiride	5–150 ng mL^–1^	5 ng mL^–1^	2 ng mL^–1^	blood plasma	(35)
LC-MS/MS	levosulpiride	3–2400 ng mL^–1^	3 ng mL^–1^		blood plasma	(36)
RP-HPLC	levosulpiride	50–150 g mL^–1^	0.20 g mL^–1^	0.06 g mL^–1^		(37)
RP-HPLC	levosulpiride	20–100 μg mL^–1^	2.8724 μg mL^–1^	0.9479 μg mL^–1^		(38)
HPLC	levosulpiride	0.965–5.790 ppm	0.386 ppm	0.965 ppm		(39)
UV spectrophotometry	levosulpiride	15–90 μg mL^–1^	3.1198 μg mL^–1^	1.0295 μg mL^–1^		(40)
			5.4627 μg mL^–1^	1.8027 μg mL^–1^		
UV spectrophotometry	levosulpiride	2–40 μg mL^–1^	0.60 μg mL^–1^	0.199 μg mL^–1^		(41)
RP-HPLC	levosulpiride	75–375 μg mL^–1^	5.517 μg mL^–1^	1.8208 μg mL^–1^		(42)
UV-spectroscopy	levosulpiride	14–84 μg mL^–1^	0.478 μg mL^–1^	0.234 μg mL^–1^		(43)
spectro fluorometry	levosulpiride	0.239–3.44 μg mL^–1^	0.0154 μg mL^–1^	0.005 μg mL^–1^		(17)
		0.310–2.730 μg mL^–1^	0.0096 μg mL^–1^	0.0032 μg mL^–1^		
SWV	levosulpiride	100–0.03 μM	2.6 nM	0.7 nM	blood plasma	this work

#### 3.5.2 Reproducibility and Repeatability of the Designed Sensing Platform

To evaluate the repeatability of the COOH-*f*MWCNTs/GCE, square wave voltammograms were obtained at different time intervals under previously optimized conditions. The results illustrated in Figure S3A indicate that the COOH-*f*MWCNTs/GCE demonstrated repeatability, reflecting the effectiveness of the proposed sensor for the electrooxidation of levosulpiride. Additionally, the reproducibility of the developed sensing platform for levosulpiride was assessed by testing various COOH-*f*MWCNTs-modified GCEs under the same optimized conditions, as depicted in Figure S3B. The SWV recordings showed no variation in the anodic peak current, confirming the excellent repeatability of the COOH-*f*MWCNTs/GCE for the electrochemical detection of levosulpiride.

#### 3.5.3 Recovery in Human Serum

The accuracy and precision of the sensing platform were evaluated by recording SWVs of levosulpiride in human serum. Initially, SWVs of human serum were recorded, and tests revealed no signs of levosulpiride. Afterward, the standard solution of the blood was prepared by collecting it from a healthy volunteer. To remove protein scums from the blood sample, 3.5 mL of acetonitrile, 3.5 mL of blood, and 2 mL of levosulpiride (2 μM) were taken in a 15 mL tube and centrifuged at 5000 rpm for 20 min. The supernatant liquid was then filtered using a 0.22 μm pore size membrane. The solution was kept in a refrigerator at 4 °C when it was not in use. The recovered amount of levosulpiride was determined from the calibration plot. After that, three readings of the as-prepared sample were performed, and the anodic peak current was matched with the *I*_p_ displayed in the calibration plot. The sensing platform demonstrated a high sensitivity toward detecting levosulpiride in blood, as validated by its relative standard deviation (RSD) of 1.55%. The targeted analyte’s percentage recovery was 85% in human blood, as depicted in Figure 5D.

#### 3.5.4 Influence of Interfering Agents

Interfering agents (I.A.) can significantly impact the detection and analysis of drug residues. The most prevalent metal ions (Pb^2+^, Cr^3+^, Cd^2+^, Hg^2+^, and Na^+^) substantially discharged by industries are identified in wastewater. These metal ions can distort the analysis by producing false-positive or false-negative results. To ensure the selectivity of the designed sensor, the interfering agent study is necessary to obtain accurate results. The impact of interfering agents on the peak current of the analyte was evaluated by simulating realistic scenarios, wherein 0.1 mM of different metal ions was introduced individually to a solution of 100 μM levosulpiride. Figure S5 illustrates the SWVs of 100 μM levosulpiride obtained in the presence of various interfering agents. The results indicated that the SWV signal of levosulpiride remained largely unaffected by significantly higher concentrations of interfering agents (RSD = 3.89%). The results can be attributed to the strong attraction of the analyte to the sensing scaffold relative to the interfering substances, along with the limited solubility of the electrode modifier in water. In light of all of the data presented above, it can be concluded that the proposed sensor has the capability of selectively detecting levosulpiride up to nanomolar concentration values.

### 3.6 Adsorption of Levosulpiride

The adsorption of levosulpiride was achieved using a polyacrylamide hydrogel. Conditions such as the adsorbent concentration, pH of the drug solution, temperature, and volume of the drug solution were optimized to achieve the best outcomes for levosulpiride adsorption. UV–vis spectroscopic monitoring was employed for the adsorptive removal study of levosulpiride. A calibration plot was necessary for accurately converting readings to concentration values. This was done by establishing a reliable correlation between the adsorbate concentration and the signal. To ensure the accuracy of quantitative analysis, the instrument’s response must be linear; otherwise, it should undergo a series of checks to address any anomalies.

**Figure 6:**
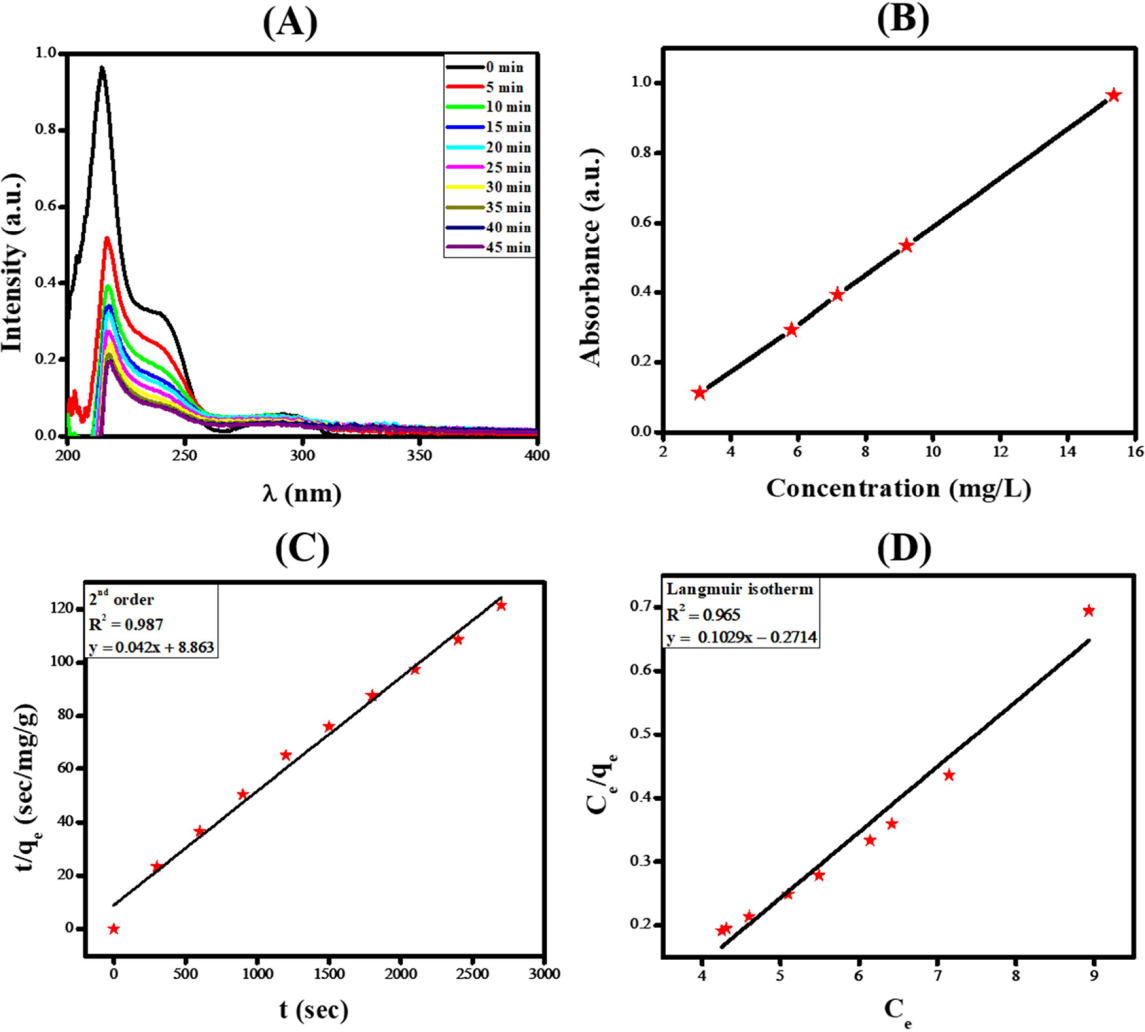
(A) UV–vis spectra showing a decrease in optical absorbance due to adsorptive removal of levosulpiride by polyacrylamide hydrogel. (B) Calibration plot of absorbance vs concentration. (C) Kinetic model for the adsorption of levosulpiride using second order kinetics. (D) Langmuir isotherm.

The calibration plot, as shown in Figure 6B, was used to convert the absorbance of levosulpiride into its concentration (ppm) after UV–vis spectra were acquired at regular time intervals during the adsorption investigations. The following equation was used to determine the maximum drug removal efficiency of the synthesized polyacrylamide hydrogel.^44^
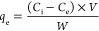
5

The equilibrium rate of drug adsorbed per unit mass of adsorbent (*q*_e_) is determined by this equation. In this case, *C*_e_ denotes the equilibrium drug concentration, *V* denotes the volume of the drug solution, and *W* is the weight of the adsorbent, while *C*_i_ stands for the starting concentration of the drug. This equation is essential for water treatment process optimization since it measures the efficiency with which an adsorbent removes impurities from the adsorbate; this measure is essential for adsorption investigations.

Several kinetic models were employed to investigate the mass transfer mechanism between the liquid and solid phases. Similarly, various adsorption isotherms were used to better understand the adsorption mechanism. Explanations of the adsorption studies are provided in the following graphs.

#### 3.6.1 Monitoring of Adsorption Using UV–Vis Spectroscopic Technique

To investigate the adsorption of levosulpiride, the required amount of polyacrylamide hydrogel was added to the drug solution. The reaction mixture was then covered with aluminum foil to prevent any potential photocatalytic reactions triggered by light. Under the preoptimized conditions (10 mg of polyacrylamide hydrogel, 45 min of contact time, 20 mL of dye, a temperature of 25 °C, and a neutral pH), the adsorption of levosulpiride using polyacrylamide hydrogel was monitored using UV–vis spectroscopy. To achieve this, samples of the drug solution were extracted at regular intervals, and UV–vis spectra were recorded for each sample. The UV–vis spectra showing a decrease in optical absorbance are shown in Figure 6A. Adsorption capacity at a specific time (*q*_e_) was calculated by using eq 5. Subsequent research was conducted to study the kinetics and adsorption isotherms.

#### 3.6.2 Kinetics of Adsorption

Kinetic analysis is essential in adsorption investigations, as it helps elucidate the removal rate of adsorbate from a solution and the mechanisms involved. Applying kinetic models such as the pseudo-first-order, intraparticle diffusion, and pseudo-second-order models to experimental data makes it possible to predict adsorption behavior and determine whether physical or chemical interactions, diffusion, or electrochemical processes at the surface govern the process.^45^ The equation used for pseudo-first-order kinetics is given as
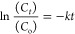
6where *t* is on the abscissa,  is on the ordinate, and *k* is the rate constant, which is equal to the value of the slope. *C*_o_ denotes the starting concentration, while *C_t_* denotes the concentration at a given time. When the adsorption rate is more affected by the availability of adsorption sites than by the concentration of the adsorbate, the pseudo-second-order kinetics equation is frequently employed to characterize the adsorption process. The equation is expressed as
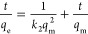
7

The abscissa represents *t*, the ordinate represents *t*/*q*_e_, the slope is 1/*q*_m_, and the intercept is 1/*k*_2_*q*_m_^2^. Maximum adsorption capacity (*q*_m_), adsorption capacity at a given time (*q*_e_), and rate constant (*k*_2_) are all represented by these variables. The intraparticle diffusion model explains how adsorbate moves through adsorbent pores. The equation for this model is given by
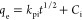
8

At a given time, the amount of adsorbate adsorbed is represented by the value *q*_e_. The intraparticle diffusion rate constant, *k*_pi_, is a measure of how quickly this adsorption process occurs. Additionally, there is a constant, *C*, related to the thickness of the boundary layer. After calculating the correlation coefficient (*R*^2^) for each of them, it was discovered that the pseudo-second-order response had the largest value. The results of our investigation showed that the adsorption process follows pseudo-second-order kinetics, as depicted in Figure 6C, where the square of the number of active sites in the sample affects the rate.

The value of *k*_2_ was determined to be 0.0002 (g/mg·s) and the adsorption capacity (*q*_m_) of 23.81 (mg/g).

#### 3.6.3 Adsorption Isotherms

The UV–vis spectroscopic data obtained from the adsorptive removal of levosulpiride using polyacrylamide hydrogel were analyzed using three different adsorption isotherm models. This analysis aimed to determine the characteristics of the adsorbent surface, such as whether it is homogeneous or heterogeneous, and to understand the interaction between the adsorbent and the drug molecules. Various isotherms were employed to evaluate the provided adsorption data. The eqs 9–11 correspond to Langmuir, Freundlich, and Temkin adsorption isotherms.
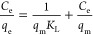
9
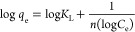
10
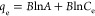
11

These variables are used to measure different aspects of the adsorption. *q*_e_ represents the adsorption capacity at a specific time, while *q*_m_ represents the maximum adsorption capacity. *K*_L_ and *K*_F_ are constants corresponding to the Langmuir and Freundlich models. The adsorption intensity is denoted by *n*, and Ce represents the concentration of levosulpiride at equilibrium. *B* is calculated as *RT*/*b*, and *A* represents the equilibrium binding constant.

The different types of isotherms were used to analyze the data collected for levosulpiride adsorption, and the data were well in agreement with the Langmuir adsorption isotherm, as shown in Figure 6D.

When an adsorption process is evaluated, the separation factor (often represented as *R*_L_) is a useful dimensionless parameter. It helps determine the favorability of the process, especially when considering the Langmuir isotherm model. It is calculated using eq 12.
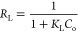
12

The type of adsorption process can be inferred from the value of *R*_L_. In the case when *R*_L_ exceeds 1, the adsorption is unfavorable. It is linear if *R*_L_ equals 1. A value of *R*_L_ between 0 and 1 is favorable. Furthermore, the adsorption is irreversible if *R*_L_ equals 0.

Based on the adsorption data, it can be concluded that the adsorption of levosulpiride onto the polyacrylamide hydrogel follows the Langmuir isotherm model. This suggests that the adsorption occurs on a uniform surface with a limited number of identical sites where each site can accommodate only one adsorbate molecule. This indicates that interactions between analyte molecules are negligible and that adsorption is primarily monolayer formation.

The strong correlation of the adsorption data with the Langmuir model implies that the polyacrylamide hydrogel has a consistent and uniform surface in adsorption sites, leading to the uniform binding of levosulpiride throughout the hydrogel. The maximum adsorption capacity (*q*_m_) derived from the Langmuir isotherm is a key measure of the hydrogel’s efficiency in removing levosulpiride from the solution. The adsorption process is also assessed using the separation factor (*R*_L_), where the value of *R*_L_ reflects the favorable adsorption of levosulpiride.

In conclusion, the adsorption process’s adherence to the Langmuir isotherm demonstrates the high effectiveness of polyacrylamide hydrogel as an adsorbent for levosulpiride, making it a highly promising material for applications such as drug removal and water treatment.

The adsorption of levosulpiride onto polyacrylamide hydrogel involves a complex process, as indicated by the data fitting both the Langmuir and Temkin isotherms. The Langmuir isotherm suggests that adsorption occurs as a monolayer on a uniform surface with evenly distributed adsorption sites. In contrast, the Temkin isotherm accounts for interactions between adsorbed molecules and a gradual decrease in adsorption energy as the coverage increases. The dual adherence to these isotherms indicates that the adsorption process involves uniform surface binding and significant interactions between the adsorbate and the adsorbent. This comprehensive insight into the adsorption mechanism highlights the effectiveness of the hydrogel in removing levosulpiride.

The values of the parameters obtained from pseudo-second-order kinetics and different isotherms are given in Table 2.

**Table 2:** Parameters Obtained from the Adsorption Data

model	parameters	abbreviation	values
kinetic parameters	rate constant	*K*_2_	0.0002 (g/mg·s)
	max. adsorption capacity	*q*_e_ (experimental)	23.81 (mg/g)
		*q*_e_ (theoretical)	30.72 (mg/g)
Langmuir adsorption isotherm	adsorption capacity	*q*_m_	9.7 (mg/g)
	Langmuir constant	*K*_L_	0.379 (L/mol)
	separation factor	*R*_L_	0.17 (unitless)
Freundlich adsorption isotherm	Freundlich constant	*K*_F_	5.636 (mg/g)
	heterogeneity	*n*	2.538 (unitless)
Temkin adsorption isotherm	Temkin constant	*b*	12.21 (J/mol)
	equilibrium binding constant	*A*	26.84 (L/g)

#### 3.6.4 Thermodynamic Parameters

Thermodynamic investigations in adsorption studies are crucial for determining the adsorption process’s feasibility, spontaneity, and nature.^46^ The Gibbs free energy (Δ*G*), enthalpy (Δ*H*), and entropy (Δ*S*) help to assess whether the adsorption is spontaneous and whether it is exothermic or endothermic. These parameters were analyzed at different temperatures (293, 298, 303, 308, and 313 K), and the corresponding values are provided in Table 3. A positive Δ*H* indicates that the process is endothermic, while a negative Δ*H* suggests that the process is exothermic. Thermodynamic studies also offer insights into the randomness at the solid-solution interface and the energy changes during adsorption, which are essential for optimizing conditions to achieve the maximum efficiency and stability of the adsorption process.

**Table 3:** Thermodynamic Parameters

				–Δ*G*^o^ (kJ/mol)				
sorbate (mg/L)	sorbent (mg)	Δ*H*^o^ (kJ/mol)	Δ*S*^o^ (kJ/mol)	293 K	298 K	303 K	308 K	313 K
0.02	0.01	0.744	0.038	10.39	10.58	10.77	10.96	11.15

Thermodynamic parameters were calculated using van’t Hoff's equation.^47^ By plotting  vs 1/*T*, the value of Δ*H* can be determined from the slope, while Δ*S* can be determined from the intercept. *R* symbolizes the general gas constant (8.314 kJ/mol), while *T* is the absolute temperature in Kelvin (K).
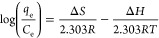
13
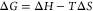
14

The value of Δ*S* calculated using the van't Hoff plot was positive (0.038 kJ/mol), indicating an increase in the disorder of levosulpiride at the adsorbate–adsorbent interface. The value of Δ*G* calculated from the Gibbs equation is negative at all of the studied temperatures, confirming the spontaneity and feasibility of the adsorption process. Additionally, the positive value (0.744 kJ/mol) of Δ*H* confirms the endothermic nature of the adsorption. Additionally, the value of the equilibrium constant can be determined using the following equations.
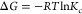
15
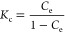
16

The value of *K*_c_ determined from eq 16 is −1.30, which is negative and greater than 1, confirming the reaction’s spontaneity.

## 4 Conclusions

The COOH-*f*MWCNTs and polyacrylamide hydrogels were successfully synthesized and characterized. Results revealed the effectiveness of polyacrylamide for the adsorptive removal of levosulpiride drug. The electrochemical role of the synthesized materials for electrode modification was explored, and the results revealed that COOH-*f*MWCNTs/GCE is an effective platform for levosulpiride sensing. Optimal experimental conditions were established by using square wave voltammetry, resulting in remarkable sensitivity with a detection limit of 0.7 nM for levosulpiride. The cyclic voltammetry and impedance spectroscopy findings revealed that the electrode modifier enhances the electroactive surface area and minimizes the charge transfer resistance, as required for efficient electron transport. The sensor demonstrated excellent repeatability, reproducibility, and selectivity, indicating its ability for practical application. The adsorptive removal of levosulpiride using a polyacrylamide hydrogel was investigated through UV–vis spectroscopy. The polyacrylamide hydrogel achieved a 78% removal of levosulpiride in just 45 min, according to the pseudo-second-order kinetics. Kinetic and thermodynamic analyses confirmed that the adsorption process is efficient and spontaneous.

## Supporting Information Available

Parameters obtained from CV and EIS analysis; Randle’s equivalent circuit; plots related to the supporting electrolyte, deposition potential, and deposition time; SWVs of repeatability and reproducibility; plots of different models and isotherms applied on adsorption data; and influence of the interfering agent (PDF)
