# Supplementary material for: Electrochemical Sensor for Levosulpiride Detection and Its Adsorptive Removal from Wastewater
Source: ACS Omega. 2025 Apr 23;10(17):17529–42. doi: 10.1021/acsomega.4c11218 (PMC12060037; doi:10.1021/acsomega.4c11218)
Supplement: Supplementary file 1 — ao4c11218_si_001.pdf [file ao4c11218_si_001.pdf]

Electrochemical sensor for levosulpiride detection and its adsorptive removal from wastewater

Mohsin Javed[a], Afzal Shah[a]*, Iltaf Shah[b]*

[a]Department of Chemistry Quaid-i-Azam University, Islamabad 45320, Pakistan

[b]Department of Chemistry, College of Science, United Arab Emirates University, Al Ain P.O. Box 15551, United Arab Emirates

Correspondence*: afzals_qau@yahoo.com (A.S.); altafshah@uaeu.ac.ae (I.S.)

Supporting Information

Table S1: Parameters obtained from CV analysis

Working Electrode	Surface Area (cm^2)	Peak Separation (ΔE_p) (mV)	Exchange Current Density (J_o) ($\mu A/cm^2$)
Bare GCE	0.037	107	5.51
COOH-*f*MWCNTs/GCE	0.086	69	55.84

Table S2: Parameters obtained from EIS analysis

Working Electrode	R_s (Ω)	R_{ct} (Ω)
Bare GCE	138	4653
COOH-*f*MWCNTs/GCE	130	459.7

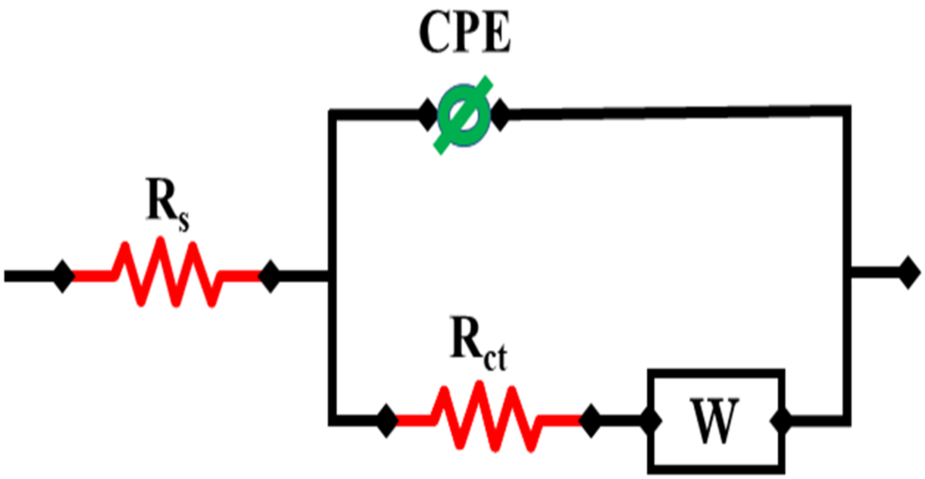

Figure S1: Randel's equivalent circuit for the assessment of EIS parameters.

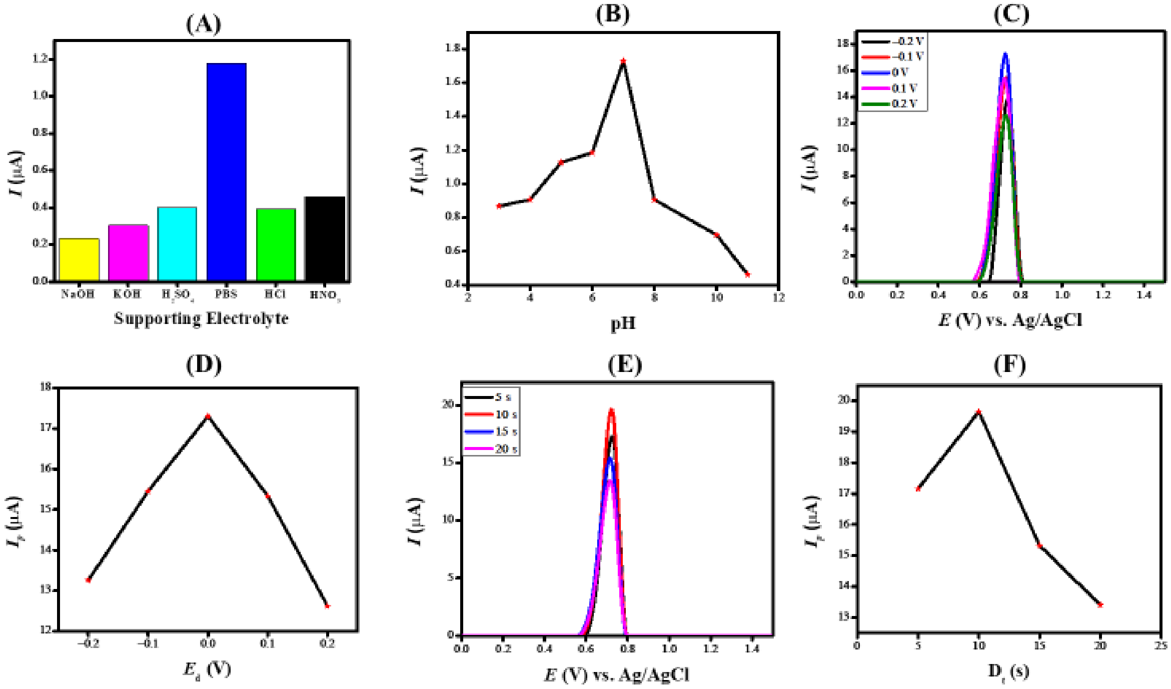

Figure S2: (A) Bar graph showing influence of supporting electrolyte on anodic peak current of levosulpiride **(B)** Plot showing variation of peak current as a function of pH **(C)** Impact of deposition potential on peak current of levosulpiride using PBS (pH 7) as a supporting electrolyte **(D)** Peak current variation in relation to deposition potential of levosulpiride **(E)** Influence of deposition time on peak current of levosulpiride at a deposition potential of 0 V **(F)** Plot of I_p vs. D_t.

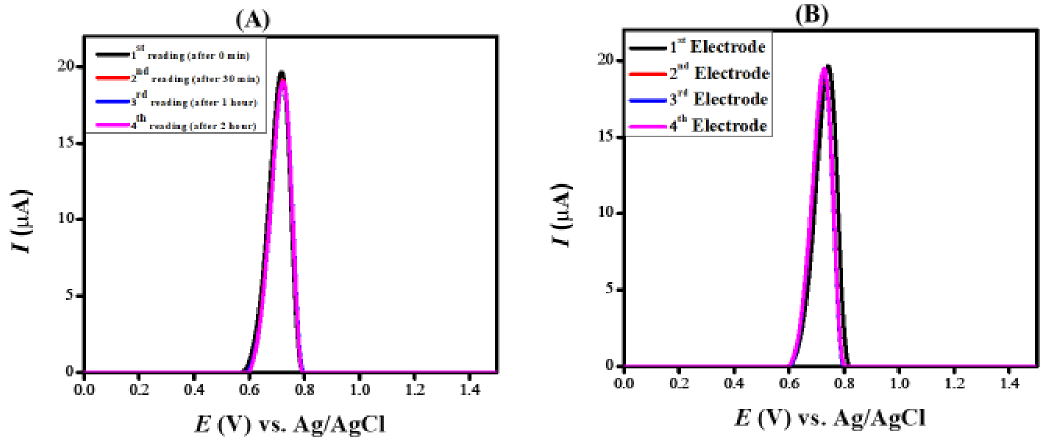

Figure S3: (A) SWVs showing repeatability for electro analysis of levosulpiride **(B)** SWVs showing reproducibility for electro analysis of levosulpiride.

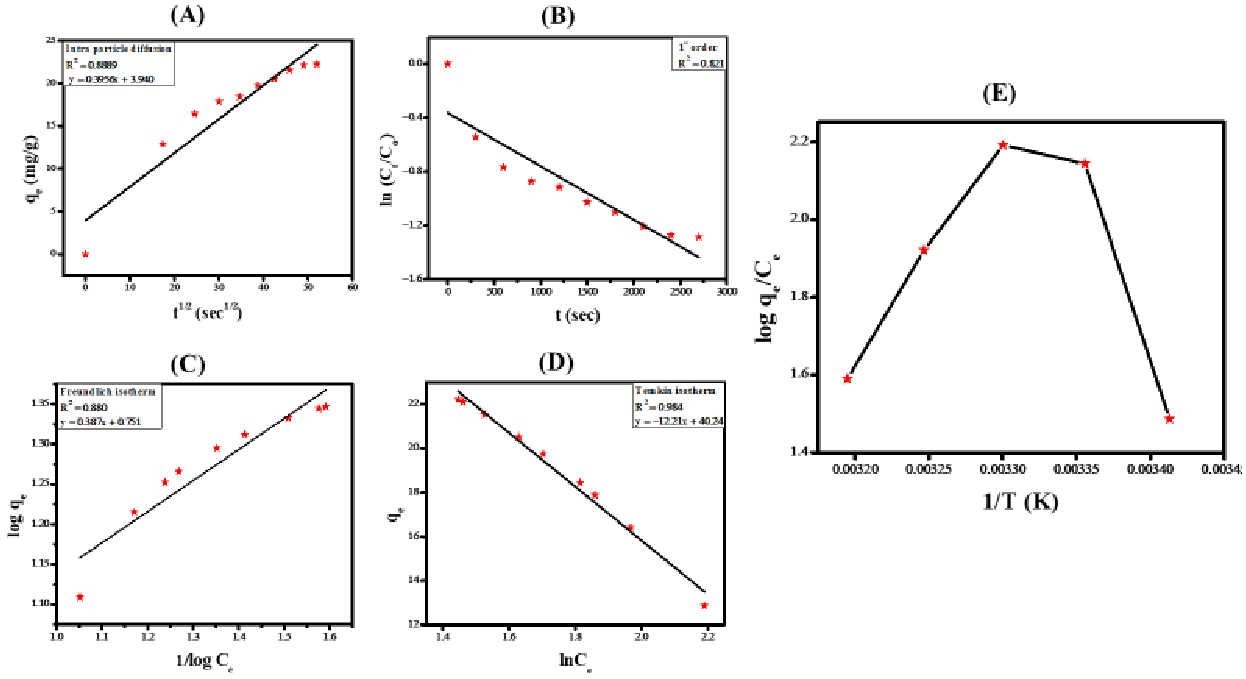

Figure S4: (A) Intra-particle diffusion model **(B)** 1st order kinetics **(C)** Freundlich isotherm **(D)** Temkin isotherm **(E)** Vant' Hoff plot

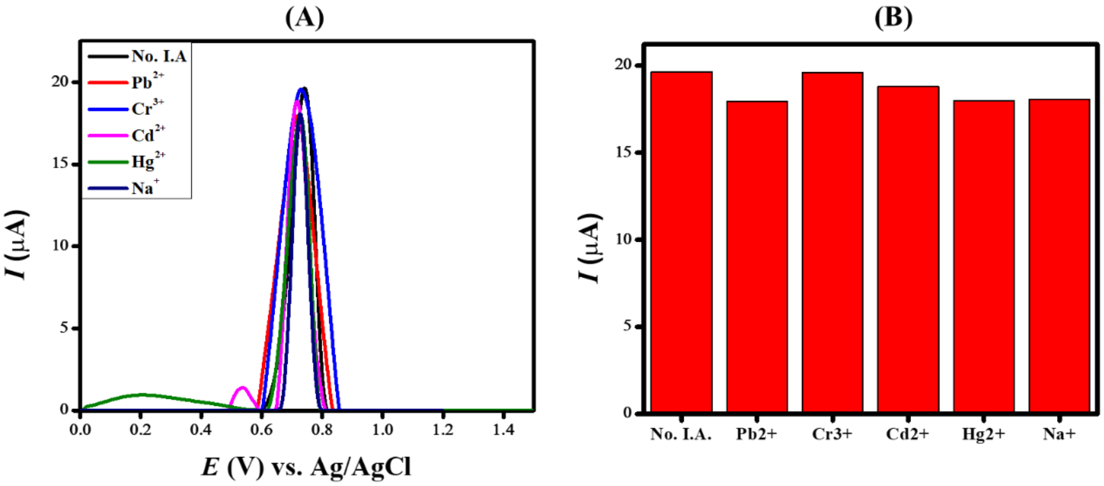

Figure S5: (A) SWVs of 100 μM levosulpiride obtained at the designed sensor (COOH-*f*MWCNTs/GCE) under optimized conditions in the presence of various co-existing interfering agents **(B)** Bar graph showing a comparison of the anodic peak intensity of 100 μM levosulpiride in the absence and presence of interfering agents.
